# Differences in senescence of late Endothelial Progenitor Cells in non-smokers and smokers

**DOI:** 10.18332/tid/135320

**Published:** 2021-06-02

**Authors:** Kumboyono Kumboyono, Indah Nur Chomsy, Wiwit Nurwidyaningtyas, Fibe Yulinda Cesa, Cholid Tri Tjahjono, Titin Andri Wihastuti

**Affiliations:** 1 School of Nursing, Faculty of Medicine, University of Brawijaya, Malang, Indonesia; 2 Faculty of Medicine, University of Brawijaya,Malang, Indonesia; 3 Department of Cardiology, Faculty of Medicine, University of Brawijaya, Malang, Indonesia; 4 Department of Basic Nursing Science, Faculty of Medicine, University of Brawijaya, Malang, Indonesia

**Keywords:** progenitor cells, late EPCs, EPC dysfunction, senescence, smoking

## Abstract

**INTRODUCTION:**

Endothelial Progenitor Cells (EPCs) are part of hematopoietic stem cells that differentiate into endothelial cells during their blood vessels’ maturation process. The role of EPCs is widely known to contribute to repair of the vascular wall when endothelial dysfunction occurs. However, various risk factors for cardiovascular disease (CVD) influence EPC performance, leading to endothelial dysfunction. One EPC dysfunction is decreased amount of EPC mobilization to the injured tissue. EPC dysfunction reduces the angiogenetic function of EPCs. The vital maturation process that the EPCs must pass is the late phase. The dysfunction of late EPCs is known as senescence. This study aimed to identify and compare senescence of late EPCs, through CD62E and CD41 markers, in non-smokers and smokers as a risk factor for CVD.

**METHODS:**

EPC collection was from peripheral mononuclear cells (PBMCs) in non-smokers (n=30) and smokers (n=31). The EPCs were then marked by CD62E/CD41 and senescence β-galactosidase assay using FACS. Identification of senescence cells was based on fluorescence with DAPI.

**RESULTS:**

Positive percentage of late EPCs in non-smokers was not significantly different from that in smokers (p=0.014). The number of senescent late EPCs in smokers was higher than in non-smokers (p<0.0001).

**CONCLUSIONS:**

Endothelial progenitor cells that experienced senescence in the smokers showed EPC dysfunction, which resulted in decreased cell angiogenic function. Further research is needed to explain the mechanism of re-endothelialization failure in EPC dysfunction due to smoking.

## INTRODUCTION

The endothelium is a unique structure in the vascular connection between the blood circulation and several organ systems. This layer is composed of endothelial cells. Endothelial dysfunction is one of the earliest events in the pathogenesis and prognosis of cardiovascular disease^1,2^. Endothelial dysfunction is often caused by long-term exposure to CVD risk factors. When experiencing interference, endothelial cells will be released from the bottom of the membrane layer and circulate into the bloodstream, known as circulating endothelial cells (CEC)^3^. Endothelial cells that are damaged or released into the bloodstream, as in non-smoker endothelial cells, will replace endothelial cells’ function. However, if the number of damaged endothelial cells is large, the endothelial cells will need help from progenitor cells produced from bone marrow^4-6^.

Endothelial Progenitor Cells (EPCs) are part of hematopoietic stem cells (HSCs) derived from peripheral blood isolation or umbilical cord isolation. EPCs have received much attention from researchers because EPC number and function indicate the severity of endothelial dysfunction. However, the isolation of EPCs is highly dependent on the source of the EPC isolation, the culture method, and the surface marker used^5,7^. In another study, two types of EPCs were isolated *in vitro* from peripheral blood vessels and other mononuclear cells, namely early EPCs and late EPCs. EPC shape and characteristics can only be determined by *in vitro* studies based on the length of time observed on culture media. Early EPC cells are spindle-shaped with limited proliferation capabilities; in contrast late EPCs are shaped with enormous reproduction capacities^8,9^.

Late EPCs result from the continuation of the maturation phase, which were previously the early EPCs. Late EPCs are an essential phase because it is at the end of EPC maturation before differentiating into endothelial cells. Dysfunction in this phase causes EPCs not to differentiate into endothelial cells. Senescence is a late EPC dysfunction that fails to saturate, so that EPCs cannot differentiate and perform their function^10-12^. Circulating late EPCs (CEPCs) play a role in the reendothelialization process in the damaged endothelial area, which releases paracrine signals to help EPCs break into the trans-endothelial layer. Specific surface markers can differentiate EPCs and other cells. Common surface markers for late EPCs are VE-cadherin (CD144), platelet endothelial cell adhesion molecule-1 (CD31), endothelial NO synthase, E-selectin (CD62E) and von Willebrand factor (VWF) (CD41)^3^. Previous studies have shown that the late EPC phase identification process *in vitro* takes 2–3 weeks with the VE-cadherin marker and the VWF marker^5^.

Changes in the population of late EPCs in circulating blood are often associated with vascular function changes even when no symptoms appear. In general, the relationship between each risk factor’s effect on the population of late EPCs is still unknown because CVD consists of more than one risk factor, for example, smoking, hypertension, diabetes mellitus, and ureteritis with a history of chest pain. Smoking is the easiest risk factor to measure because smoking is a very influential initial risk factor and can be practised by people without any symptoms^4,6,13^. In this study, researchers characterized late EPCs directly from peripheral blood mononuclear cells in adults without going through the culture stage. This study aimed to identify and compare senescent late EPCs through CD62E and CD41 markers in non-smokers and smokers. This study’s significance is to prove the occurrence of senescence in the late phase EPCs as an early screening for the initial endothelial dysfunction in smokers.

## METHODS

### Sample of the study

Peripheral mononuclear cells (PBMCs) were obtained from non-smokers (n=31) and smokers (n=30). Researchers obtained research participants after announcing participant recruitment via social media for two weeks. Non-smokers met the inclusion criteria: aged 20–40 years, did not smoke, had no history of diabetes mellitus, dyslipidemia, and hypertension. The smokers were aged 20–40 years, daily smokers, no history of diabetes mellitus, dyslipidemia, and hypertension.

Researchers checked participants’ age suitability by checking each subject’s official identity cards. Nonsmoker status is determined based on the results of a semi-quantitative determination of cotinine in saliva (an immunochromatographic assay that uses monoclonal antibody-coated gold particles and a series of avidity traps that allow quantification (Confirm Biosciences Inc, San Diego, California, USA). Participants were confirmed as non-users of tobacco products if they had a value of 0–10 ng/mL of cotinine concentration. Meanwhile, daily smoker status was determined by Fagerström nicotine dependence test with score 4–6 (medium tobacco dependence).

The absence of a history of diabetes mellitus and dyslipidemia was obtained from the history and examination of the participants’ venous blood at Saiful Anwar General Hospital’s central laboratory unit. The participants’ glucose levels were confirmed by examining fasting blood glucose and haemoglobin A1c (HbA1c) levels. Measurement of fasting blood glucose used enzymatic reference method with hexokinase, while HbA1c levels used high-performance liquid chromatography. A fasting blood glucose level of <100 mg/dL (5.6 mmol/L) is standard. The average value for HbA1c is between 4% and 5.6%. The analytical method for calculating cholesterol uses an enzymatic assay with expected values for adults of <200 mg/dL.

Researchers confirmed no history of hypertension by taking anamnesis and measuring blood pressure using a mercury blood pressure monitor (Riester Nova Ecoline, Germany). The participants in a lying position (average score after three measurements, after 5 minutes break). The researchers standard for the mean blood pressure level was 110–120 mmHg for the systolic and 70–80 mmHg for the diastolic.

### PBMCs isolation

A volume of 10 mL of PBMCs was taken using an EDTA vacutainer, 5 mL of lymphoperm and 5 mL of PBS were added then centrifuged at 1600g for 45 minutes at room temperature. The buffy coat layer was taken and washed three times with PBS, then resuspended with Cell Suspension Buffer (CSB) as much as 50 µL.

### Late EPC isolation

The cell suspension was added with marker staining CD62E anti-human Phycoerythrin (PE) no.cat.22606 (Biolegend) and peridinin chlorophyll protein complex (PerCP) / cyanine 5.5 anti-human VWF / CD41 no.cat.30372 (Biolegend), respectively, as much as 5 µL, and then incubated at room temperature for 20 minutes.

### Senescence assay

The cell suspension was again centrifuged at 4°C and 6000g for 5 minutes. The supernatant was discarded, then 100 µL of paraformaldehyde was added and incubated for 10 minutes.

Cellular ageing was performed using CellEvent™ Senescence Green Flow Cytometry Assay Kit Brand: Invitrogen No. Paint. 10841, with a total concentration of 1:500. After washing with CSB, it was incubated for 2 hours at room temperature 37°C without CO_2_. Total cell count and percentage of β-galactosidase-positive cells were calculated by the BD FACS Melody analyzer (USA).

### Late EPC fluorescence

Senescence cells were identified using a twodimensional microscope (Olympus IX71) at the Biomedical Laboratory, Faculty of Medicine, Universitas Brawijaya. After staining the senescence antibody assay, the cell suspension was centrifuged at 2500g and 4°C for 5 minutes. The supernatant was discarded, 200 µL of DAPI (1:1000) were added, then incubated in a dark room for 5 minutes, and then centrifuged, washed with CSB, and the pellets placed on the glass object for observation using a microscope with 400× magnification. Senescence cells are identified as a greenish color.

### Data analysis

The number of late EPCs and senescence assay were analyzed using the independent t-test, and the values were expressed in terms of mean ± SD. The p-value of <0.05 was considered as significantly different.

### Ethical clearance

Informed consent was obtained from all study participants, and the study was approved by the Ethics Commission of the Faculty of Medicine, Brawijaya University Malang (No. 149/EC/KEPK/08/2020).

## RESULTS

Table 1 indicates that the two groups of research subjects have homogeneous characteristics. Nonsmokers and smokers have the same age and sex proportions. Likewise, both groups did not have a history of diabetes mellitus, dyslipidemia, and hypertension.

**Table 1:** Characteristics of the participants of the research

*Variables*	*Smoking status groups*		*p*
	*Non-smokers (n=31)*	*Smokers (n=30)*
**Age** (years), n (%)			0.632*
21–30	10 (32.26)	8 (26.67)	
31–40	21 (67.74)	22 (73.33)	
**Sex**, n (%)			0.900*
Male	16 (51.61)	15 (50)	
Female	15 (48.39)	15 (50)	
**Fasting blood glucose** test (mg/dL), mean±SD	88.19±6.16	90.17±6.77	0.238**
**Hemoglobin A1c levels** (%), mean±SD	4.63±0.38	4.75±0.37	0.205**
**Systolic Blood Pressure** (mmHg), mean±SD	116.45±4.86	116.00±4.98	0.721**
**Diastolic Blood Pressure** (mmHg), mean±SD	76.45±4.86	76.0±4.98	0.722**
**Total Cholesterol** (mg/dL), mean±SD	137.65±20.89	137.80±16.01	0.974**

* Calculated using chi-squared at a level of significance of 5%.

** Calculated using the independent t-test at a level of significance of 5%.

**Figure 1:**
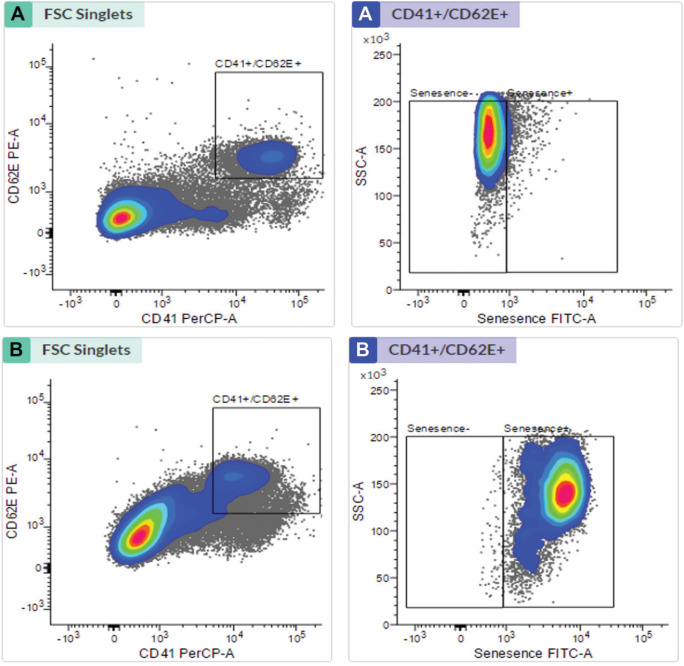
Late EPC analysis based on FACS Melody analyzer: (A) Late EPC population in non-smokers and (B) Late EPC population in smokers

Table 2 shows from the results of EPC identification that the number of late EPCs found in the smokers was not significantly different from that of the non-smokers (p=0.014). Based on observations, the number of late EPCs that experienced positive senescence was higher in smokers than in nonsmokers (p<0.0001). In contrast, the number of late EPCs with negative senescence found in the smokers was lower than in the non-smokers (p<0.0001). Box plot results obtained, describe the distribution of data from the isolation and senescence assay using FACS (Figure 1).

**Table 2:** Comparison positive percentage of late EPC between non-smokers and smokers

*Variables*		*Smoking status groups*		p*
		*Non-smokers (n=31) mean±SD*	*Smokers (n=30) mean±SD*	
Positive percentage of	Late EPC population	7.012±3.243	9.031±2.978	0.014
	Late EPC expressing senescence	8.103±6.744	98.151±0.755	<0.0001
	Late EPC not expressing senescence	90.497±7.106	1.044±0.632	<0.0001

* Calculated using the independent t-test at a level of significance of 5%.

**Figure 2:**
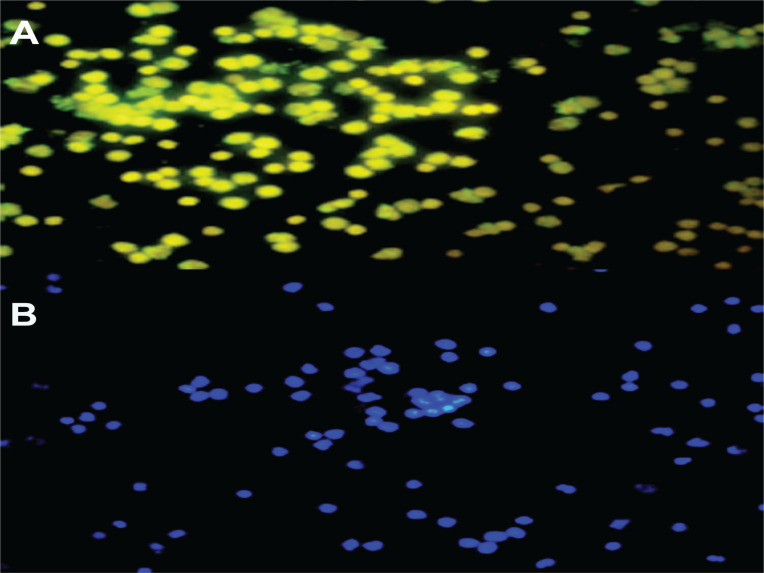
Late EPC cell fluorescence depicting cell populations experiencing senescence with 400× magnification

Late EPC analysis results for the non-smokers and smokers were based on CD41 (von Willebrand Factor /VWF) and CD62E (endothelial NO synthase, E-selectin) markers as indicators of late EPCs. Positive senescence is a late EPC expressing Senescence-Associated-β-galactosidase (SA-β-gal). Based on Figure 1, the number of late EPCs are higher in the smokers than in the non-smokers. In this study, positive senescence contained contrasting blue to red areas, indicating that late EPCs experienced more senescence in the smokers than in the non-smokers. The observation of fluorescence results was confirmed using fluorescein isothiocyanate and SA-β-gal and nucleus staining using 4,6-diamidino-2-phenylindole (Figure 2). Based on the results of fluorescence observations, the cells that express SA-β-gal in the smokers are more than the cells that do not express this marker.

## DISCUSSION

The study of EPCs is quite interesting because of its role in the pathogenesis of atherosclerotic diseases such as coronary artery disease (CAD)^14^. In this study, data were presented related to senescence from the late EPC senescence phase, which is thought to have a relationship to EPC dysfunction. EPC dysfunction takes the form of premature ageing in the late EPCs, causing paracrine signal transfer failure and failure to invade the damaged vascular area^15^. The results of this study indicate the number of late EPCs isolated with the marker CD41+/CD62E+ and the cells experiencing senescence with the β-galactosidase marker in the non-smokers and the smokers. The parameters observed (Table 1) in each group were the late EPCs, the number of senescence cells, and the number of cells without senescence, which generally distributed homogeneously. The table shows that the late EPCs and cells experiencing senescence were more in the smokers.

This study shows numerous late EPCs found in the smokers. These cells might be released from bone marrow and involved in the differentiation process towards the trans-endothelial layer due to a stimulus in the form of oxidative stress^16^. EPCs differentiate when circulating in the blood and begin when hematopoietic stem cell markers such as CD133 and CD117 gradually disappear and are replaced by the endothelial-like phenotype^17^. EPCs take a long time to repair damaged endothelium, so it takes a sufficient number of EPCs to mobilize to reach the target network, this then becomes a quality parameter of the EPCs^18^. Cells are affected by many internal and external stimuli, some of which can induce stress that results in damage to the structure and function of proteins, DNA, or other essential macromolecules. The cell’s capacity to respond to stress depends on the species and type of cell and the proteome that is expressed over a specific time^19^. If a lot of stressor stimulation influences the late EPCs, the cells will experience a change in structure/function in the form of senescence.

Late EPC dysfunction in the smokers can be seen from the significant difference in the number of late EPCs experiencing senescence than in the non-smokers. The difference in the late EPCs senescence in the two groups indicated EPC dysfunction in the smokers in the late phase. EPCs that come from bone marrow during the production/repopulation will experience maturation until the late phase as a final indicator of EPCs readiness to differentiate into endothelial trans and replace endothelial cells that have lost their function^3,16^. Over time, senescent cells will lose their essential biological function. The cell cycle from the late EPCs will normally run again when the microenvironment’s stressor decreases. The accumulation of stressors can cause late EPCs that experience senescence to experience cell cycle cessation, thus inducing the pathway apoptosis mechanism^11,12^. Cell senescence not only results in endothelial dysfunction but further induces failure of angiogenesis. EPCs senescence may not be associated with telomere shortening as a common cause of cellular senescence. Still, there is a tendency for EPCs senescence to occur due to increased expression of p-16 cell cycle inhibitors via the SIRT-1 pathway^20^. The increase in p16 expression in the cell ageing process will be physiologically linear with increasing age. However, there is no further explanation regarding the relationship between the appearance of p16 expression, starting at old age, or whether it has been around since atherogenesis in the second decade. Another study suggested that the involvement of p16 in these ageing progenitor cells was uncertain. Still, stem cells were known to exhibit low internal replication ability and were highly susceptible/ responsive to stress-induced premature senescence (SIPS)^9^. We know that processes, such as smoking, can trigger oxidative stress in cells, one of which is EPCs experiencing senescence and failure to carry out their function related to reendothelialization.

The cellular senescence marker used in this study was the SA-β-gal. Several researchers have discussed cellular senescence markers from various perspectives in smokers. Senescent cells display an enlarged and flattened cell shape^15^. Elevated SA-β-gal activity remains the gold standard to identify senescent cells in culture and tissue samples^21,22^. Senescence-associated heterochromatin foci (SAHF) is a marker that locks cells in a senescent state by repressing genes involved in cell proliferation^23-25^. Besides, markers to lamin B1, the inner nuclear membrane protein lamin B receptor (LBR) and the lamina-associated polypeptide-α (LAP2α), are also downregulated in senescent cells^26,27^. The research results on senescence markers reinforce this study’s outcome of EPC dysfunction in smokers.

The number of EPCs in the smoking group that we observed is not an answer to the function of vascular integrity because almost all EPCs in the smoking group have experienced senescence as part of a form of endothelial dysfunction. This research did not conduct *in vitro* measurements to validate the smoking group’s EPC senescence marker. However, *in vitro* estimation of EPC dysfunction can be confirmed by a decrease in cell proliferation^28^. *In vitro* measurements of senescence markers from the cells can be done through analysis of the ability of EPCs homing on implanted matrigel plugs or endothelial tube formation, indicating reendothelialization^29,30^. EPC dysfunction contributes to decreased angiogenic ability, which is inducted in vascular complications and atherogenesis^31,32^. The number of late EPCs can be used as a marker of vascular function to predict increased cardiovascular risk^33^.

## CONCLUSIONS

Endothelial progenitor cells that experience senescence in smokers showed EPC dysfunction, which resulted in decreased cell angiogenic function. Further research is needed to explain the mechanism of re-endothelialization failure in EPC dysfunction due to smoking.
